# Polycystic pancreatic disease associated with pineal cyst in an adolescent: a case report and literature overview

**DOI:** 10.1002/ccr3.1167

**Published:** 2017-09-05

**Authors:** Abdul‐Monem Badran, Anatoli Fotiadou, Simon Kayemba Kay's

**Affiliations:** ^1^ Department of Paediatrics Victor Jousselin Hospital 44 Avenue du président J.F. Kennedy 28102 Dreux France; ^2^ Present address: Department of Pediatrics Sion Hospital Avenue du Grand‐Champsec 80 1950 Sion Switzerland

**Keywords:** MRI, Pancreas, Pineal gland, Polycystosis

## Abstract

Pancreatic polycystosis is one of rare causes of recurrent abdominal pain of pancreatic origin in children frequently associated with other organ's cysts which are to be searched. Association with pineal cyst is exceptional, and link between the two locations is to be elucidated. MRI is highly valuable to characterize cysts.

## Introduction

Polycystic pancreatic disease is a rare condition in adults and is usually found incidentally during surgery or at autopsy 1. It is often asymptomatic or may present with vague abdominal pain. Overall, pancreatic cysts are uncommon in children, and most often associated with inherited polycystic diseases of other organs especially kidney, liver, and spleen. Isolated polycystic disease of pancreas or disease associated with pineal cyst has not been reported earlier in children.

## Case History

A 14‐year‐old female Caucasian adolescent, in whom two cystic images (15 and 10 mm) in the pancreas were discovered on abdominal US, was referred to the pediatric GI OPD of Dreux hospital (France). She suffered from isolated upper abdominal pain for 10 days. She had an unremarkable physical examination. Her weight was 45.4 kg, height 148 cm, and her pubertal stage was Tanner 5. The parents were not consanguineous. There was no family history of disease of pancreas, kidney, liver, or cystic disease. As relevant past history, she had presented with upper abdominal pain which had lasted few days a year earlier. Upon admission in the OPD, she was free of symptoms. A treatment with a proton pump inhibitor was prescribed by the family physician.

**Figure 1:**
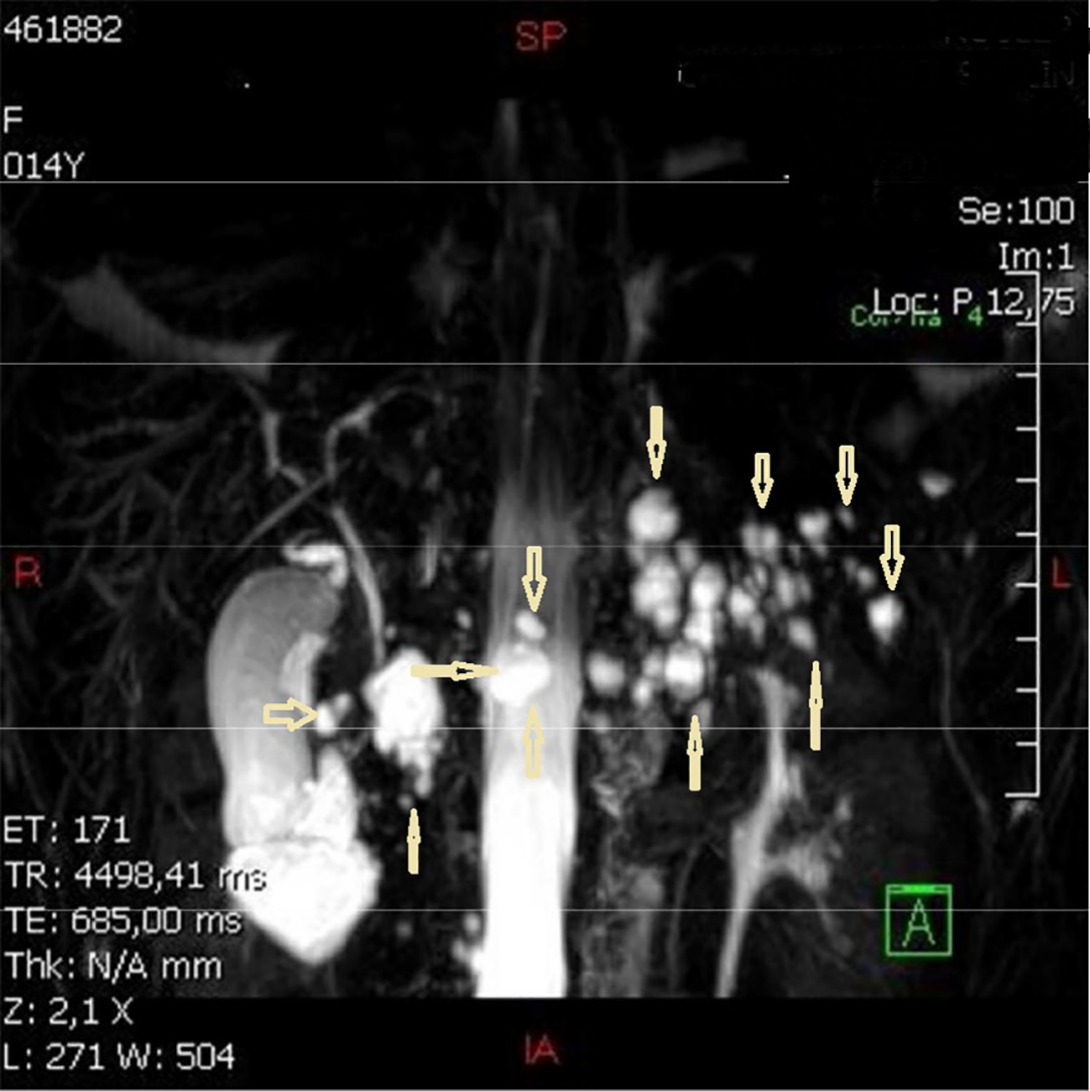
Pancreatic MRI showing a high number of pancreatic cysts of variable sizes scattered throughout the Parenchyma.

**Figure 2:**
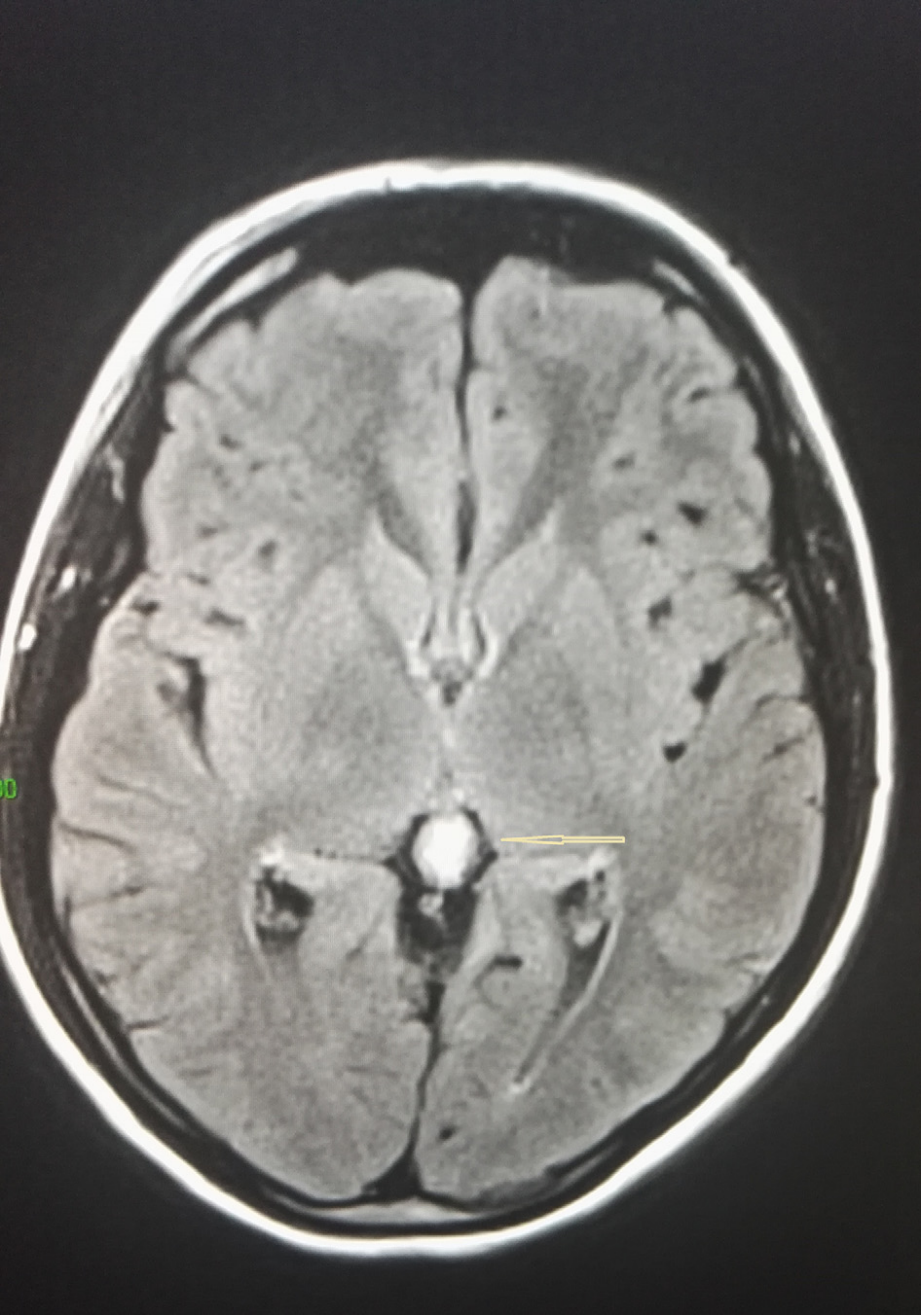
Brain MRI showing 7–8 mm pineal cyst.

Biochemical tests showed normal liver and renal functions as well as normal blood electrolytes and unremarkable full blood cell counts. Plasma lipase and amylase were normal. Serology for Echinococcus granulosus was negative. The sweat chloride test was normal. Fecal elastase, insulin, and C peptide values were normal as well as oral glucose tolerance test. Abdominal MRI revealed large number of pancreatic cysts of variable sizes throughout the parenchyma, with the biggest measuring 20 mm in diameter (Fig. 1) without evidence of hemorrhage or inflammation or other parenchymal or ductal abnormality. There were no cysts seen in the liver, spleen, or kidneys. Genetic testing for chronic hereditary pancreatitis or susceptibility genes did not detect any abnormality in CFTR (29 mutations), PRSS1, SPINK 1, or CTRC genes. Brain MRI revealed a 7 mm pineal cyst of benign aspect (Fig. 2) without related symptoms in the child. The diagnosis of polycystic pancreatic disease was retained, associated with pineal cyst. Periodic follow‐up started for surveillance.

## Comments

Pancreatic cysts can be developmental (congenital), retentional, tumoral, parasitic, pseudo, or duplication cysts 2. True congenital pancreatic cysts, even rare, are often reported in the literature as case reports 3. Most congenital cysts of pancreas are associated with inherited polycystic disease of other organs especially the kidney, liver, and spleen. Cystic fibrosis is also reported to be a case of multiple cysts of pancreas 4. Isolated pancreatic polycystosis has not been reported earlier in children to our best knowledge, neither association with pineal cysts. The head of the pancreas is favorite localization of pancreatic cysts reported in 32% of cases in one study 2. In our adolescent, the cysts were seen in the entire pancreas. Generally, these congenital cysts are asymptomatic. Symptoms as abdominal distention, vomiting, jaundice, or pancreatitis can be observed 2, 4. Concerning our patient, isolated upper abdominal pain was the revealing symptom. Multiple pancreatic cysts and microcystic cystadenomas have, occasionally, been reported in patients with Von Hippel‐Lindau syndrome 5. It is noteworthy that our patient had no family history or any other manifestation suggestive of VHL syndrome.

Abdominal MRI was of tremendous contribution to the diagnosis by revealing the number of cysts, with a yield that, was superior as it provided an excellent tissue characterization along with detailed anatomical definition of the pancreas. Pancreatic cysts require no treatment, except for symptomatic one. It is rare that surgery is required. In that case, several modalities are reported, such as coelioscopic fenestration of big ones 6.

Pineal cysts are frequently seen on brain MRIs in children with a prevalence which changes with age, more frequently in girls and with a peak in young adults found to be 2–4% of brain MRIs 7, 8. They are almost always asymptomatic and are usually incidentally identified. Rarely, hydrocephalus can result from aqueductal obstruction. In our case, incidental character of the pineal cyst cannot be concluded in the presence of the pancreatic cystic disease, although the child has no related symptoms. Association of pineal cysts with polycystic kidney disease was already reported 9. To our knowledge, association with polycystic pancreatic disease was not previously reported. In fact, although the most probable pathogenesis of cysts in our case is developmental, we have not enough arguments for whether or not there is a link between these two locations. Literature also lacks in such situation.

## Conclusion

Polycystic disease of the pancreas being very rare in children, the literature lacks information on its prevalence, etiopathogenesis, management, appropriate follow‐up modalities, and long‐term outcome. To our knowledge, association with pineal cyst was not previously reported.

## Authorship

A‐MB (GI pediatrician consultant for whom the patient was referred): conducted the work‐up until the definitive diagnosis, redacted the discussion and the key clinical message, and participated in the bibliography research. AF: redacted the case history and participated in the preliminary form of the manuscript. SKK (Pediatric consultant): participated in the bibliographic research selection, in the discussion, in conclusion, and in the manuscript review.

## Conflicts of Interest

No conflict of interests to be declared by the authors.
